# Reducing the Effects of PCR Amplification and Sequencing Artifacts on 16S rRNA-Based Studies

**DOI:** 10.1371/journal.pone.0027310

**Published:** 2011-12-14

**Authors:** Patrick D. Schloss, Dirk Gevers, Sarah L. Westcott

**Affiliations:** 1 Department of Microbiology & Immunology, University of Michigan, Ann Arbor, Michigan, United States of America; 2 Microbial Systems & Communities, Genome Sequencing and Analysis Program, Broad Institute of MIT and Harvard, Cambridge, Massachusetts, United States of America; Argonne National Laboratory, United States of America

## Abstract

The advent of next generation sequencing has coincided with a growth in interest in using these approaches to better understand the role of the structure and function of the microbial communities in human, animal, and environmental health. Yet, use of next generation sequencing to perform 16S rRNA gene sequence surveys has resulted in considerable controversy surrounding the effects of sequencing errors on downstream analyses. We analyzed 2.7×10^6^ reads distributed among 90 identical mock community samples, which were collections of genomic DNA from 21 different species with known 16S rRNA gene sequences; we observed an average error rate of 0.0060. To improve this error rate, we evaluated numerous methods of identifying bad sequence reads, identifying regions within reads of poor quality, and correcting base calls and were able to reduce the overall error rate to 0.0002. Implementation of the PyroNoise algorithm provided the best combination of error rate, sequence length, and number of sequences. Perhaps more problematic than sequencing errors was the presence of chimeras generated during PCR. Because we knew the true sequences within the mock community and the chimeras they could form, we identified 8% of the raw sequence reads as chimeric. After quality filtering the raw sequences and using the Uchime chimera detection program, the overall chimera rate decreased to 1%. The chimeras that could not be detected were largely responsible for the identification of spurious operational taxonomic units (OTUs) and genus-level phylotypes. The number of spurious OTUs and phylotypes increased with sequencing effort indicating that comparison of communities should be made using an equal number of sequences. Finally, we applied our improved quality-filtering pipeline to several benchmarking studies and observed that even with our stringent data curation pipeline, biases in the data generation pipeline and batch effects were observed that could potentially confound the interpretation of microbial community data.

## Introduction

The advent of 16S rRNA gene sequencing has revolutionized how microbial ecologists understand the bacterial and archaeal world around them [1]. Although the general approach has known limitations (e.g. low rate of evolution, lack of correlation with organism function, and variable copy number), no other molecular marker has emerged that is found in all organisms, has as low a rate of horizontal gene transfer and recombination, or has sufficient genetic information to differentiate closely related organisms. Even before the advent of next generation sequencing, the 16S rRNA gene was the most well represented gene in GenBank. Inherent in every microbial ecology experiment is the hypothesis that changes in the microbial community's structure will affect the community's function. The recent advent of next generation DNA sequencing has greatly facilitated the ability to broadly test this hypothesis. It is now possible to obtain thousands of sequences per sample using pyrosequencing for the same cost of sequencing dozens of sequences by Sanger-based sequencing technology [2]. A limitation of this approach is that it is not possible to obtain a full-length sequence of the 16S rRNA gene. To overcome this limitation, PCR primers have been designed to target one or more of the 9 variable regions within the gene; there is no region that has received universal acceptance by the field. The creation of DNA barcodes, short DNA sequences are included upstream of the PCR primer, has enabled investigators to multiplex numerous samples has enabled investigators to allocate vast sequencing resources to numerous samples [3]. Furthermore, these improvements allow for more robust experimental designs; whereas biological or technical replicates were rarely obtained using Sanger technology, it has since become expected [4].

Within the biomedical sciences, analysis of 16S rRNA genes has had a significant impact on our knowledge of novel pathogens including the causative agent of Whipple's disease [5] and has forced a reconsideration of Koch's postulates in light of molecular data [6]. It has been widely suggested that Crohn's disease, obesity, periodontitis, eczema, cystic fibrosis, and myriad other diseases affecting nearly every part of the human body are caused not by single pathogens, but by consortia of microbes. The biomedical version of the structure-function hypothesis, the dysbiosis hypothesis, suggests that alterations in the structure and stability of microbial communities can bring about changes in human health and disease [7]. To test this hypothesis on a large scale, the Human Microbiome Project (HMP), funded by the US National Institutes of Health, and MetaHit, funded by the European Commission, have pursued a number of studies to define the microbial biodiversity associated with health and disease [8], [9]. For example, the HMP recruited 300 individuals, who were sampled 2 or 3 times at 15 (men) or 18 (women) body sites with the goal of characterizing the structure and function of the normal microbiome [8]. Similar efforts are underway to address how deviations in the structure and function of the microbiome relate to disease.

In spite of great excitement to pursue novel research questions, the sequencing technology has developed at such a fast rate that there have been only modest gains in improving the quality of the raw data and in understanding how that quality affects experimental design and data interpretation. Although there is interest applying Illumina's sequencing platform to whole metagenome shotgun sequencing [10], 16S rRNA gene sequencing has primarily been performed using Roche-454's sequencing platform. This platform uses a sequencing-by-synthesis approach where flows of individual nucleotides are passed over a picotitre plate and a fluorescent signal is generated proportional to the number of times that nucleotide is incorporated. In the current GS FLX Titanium protocol, 800 flows are performed. The Roche-454 platform was originally developed for genome sequencing, which does not require stringent quality filtering measures since multiple reads are assembled to create a consensus sequence. In other words, although error rates for individual reads may be high, if sufficient reads are obtained, the error rate of the assembled genomes can be very low. In contrast, analyses of 16S rRNA gene sequences do not assemble reads. Therefore, any sequencing error will cause the sequence to be naively portrayed as arriving from a novel bacterium. This has created a debate in the environmental microbiology field over how much of the “rare biosphere” is a product of sequencing error [11]–[14]. Regardless of the outcome to the debate, investigators need to understand the quality of their data and how to reduce errors. Ultimately, downstream analyses will be influenced by these errors.

There are multiple sources of bias and error in a 16S rRNA gene sequencing survey. We define biases as a misrepresentation of the relative abundances of microbial populations in a sample and errors as a misrepresentation of an actual sequence due to PCR amplification and sequencing. The method of DNA extraction and purification, PCR primer selection and cycling conditions, actual community composition, and number of 16S rRNA gene copies per genome can all affect whether the relative abundances of 16S rRNA gene sequences being sequenced are the same as the bacterium's relative abundance in the original sample [15]–[19]. There are three primary sources of error. First, PCR polymerases typically have error rates of 1 substitution per 10^5^–10^6^ bases [20]. Second, when amplifying DNA fragments from a heterogeneous template, there is a risk of chimera formation when incomplete PCR products serve as primers to amplify related fragments; the rate of chimerism is thought to range from 5 to 45% [21]. Finally, errors are introduced in sequencing, regardless of the technology. The Roche-454 platform is known to have difficulties representing homopolymers (i.e. stretches of DNA containing the same base) and has a reported error rate of 0.0100 to 0.0200 (i.e. number of errors per total base calls) for individual sequence reads [2]. Because of their relative rates, sequencing errors and chimeras are of most concern.

Sequencing error rates have generally been measured by sequencing collections of 16S rRNA gene fragments with a known sequence (i.e. mock communities). Four general approaches have been taken to reducing sequencing errors and their effects. The first approach was to remove sequence reads that had features correlated with sequencing errors (e.g. ambiguous base calls, mismatches to primers). Huse and colleagues [12] found that if they removed any read that had ambiguous base calls, mismatches to the PCR primer, or were shorter or longer than expected, then the observed error rate was reduced to 0.0016. The second approach was to trim regions of the sequence associated with low quality scores. Kunin and colleagues [14] sequenced the 16S rRNA gene from *E. coli* and expected to observe 1 operational taxonomic unit (OTU) when clustering at a 3% cutoff, but instead observed 16. When they used LUCY to identify regions within each sequence that had an average quality score greater than 27 they obtained the correct number of OTUs [22]. The third approach was the development of two denoising algorithms, PyroNoise and DeNoiser, which correct the base calls by modeling the original flowgram data [23]–[25]; unfortunately, these methods have received limited application because they require high computational resources and have had implementations that are difficult for most investigators to use. Two less computationally demanding denoising algorithms, single linkage pre-clustering and SeqNoise, have also been developed to remove sequencing errors [13], [24]. Although these three approaches reduce the error rate, a fourth set of approaches has developed heuristics to essentially fit the observed number of OTUs to the expected number of OTUs without concern for the error rate. These have included removing sequences that cannot be taxonomically classified [26], using pairwise sequence alignments instead of a multiple sequence alignment [13], clustering sequences by the average neighbor algorithm instead of the furthest neighbor algorithm [13], using broad OTU definitions [14], and removing sequences that are below an abundance threshold has also been applied to Illumina-generated sequence data [27]. These studies generally have had the goal of reducing the number of spurious OTUs and phylotypes, not minimizing the actual error rate. This has the effect of limiting the generalizability of results to other experimental frameworks.

Although there are a number of steps that can be taken to reduce the rate of chimerism [21], [28], [29], three bioinformatic approaches have recently been developed to identify chimeras and remove them from the analysis. First, Haas and colleagues [21] developed the ChimeraSlayer algorithm, which they showed to be superior to Bellerephon [30] and Pintail [31], especially for short sequences and in cases where the parents of the chimera were closely related to each other. Second, Quince and colleagues [24] developed Perseus, which does not use a reference database, but does require a training set of sequences similar to the sequences being characterized. Finally, Edgar and colleagues [32] developed Uchime, which showed improved performance over ChimeraSlayer, especially in cases where the chimera has more than two parents; Uchime's performance was comparable to that of Perseus. As each of these studies discussed, there is a tradeoff between the specificity and sensitivity that can be modulated to serve an investigator's needs; however, the creators of the tools have emphasized specificity over sensitivity. It is important to note that chimeras are not sequencing errors and because, by definition, there is not a single reference sequence to map the chimera to, chimera frequency should be treated separately from the sequencing error rate.

As part of the Human Microbiome Project, three benchmarking studies were implemented to evaluate the implementation of a standardized operating procedure (SOP) by the sequencing centers at the Baylor College of Medicine (BCM), Broad Institute (BI), J. Craig Venter Institute (JCVI), and the Washington University Genome Sciences Center (WUGSC). First, DNA from the V13, V35, and V69 regions of the 16S rRNA gene were amplified and sequenced from a mock community representing the genomic DNA from 21 isolates. Second, DNA from the same regions of a single stool sample was PCR amplified and sequenced. For these two studies the sequencing centers performed between 1 and 5 sequencing runs and each run including three replicates for each region using the Roche 454 GS FLX Titanium platform. The goal of these two studies was to assess the robustness of the SOP by measuring bias, sequencing error rates, and intra and inter-sequencing center variation. Between the 10 total runs, there were a total of 30 mock community and stool sample replicates per region of the 16S rRNA gene and a total of 2.7×10^6^ sequences. These data are part of Project SRP002397 in the NCBI Short Read Archive. The third study sought to benchmark the SOP by performing a pilot of the full HMP 16S rRNA gene sequencing effort. The pilot study consisted of sampling 15 and 18 body sites from 12 men and 12 women, respectively. The DNA was isolated at BCM and the 396 samples were randomly sent to two of the four sequencing centers for PCR and sequencing of the V13 and V35 regions of the 16S rRNA gene. The centers attempted to obtain at least 5,000 sequence reads from each sample; there were a total of 18.7×10^6^ sequences in the dataset. These data are part of Project SRP002012 in the NCBI Short Read Archive. In the present study we used these massive datasets to create a pipeline that minimized the sequencing error rate and incidence of chimeras. We then implemented the pipeline to understand the effect of these sources of error on the interpretation of microbiome data.

## Results

### Basic characteristics of mock community sequence data

**Figure 1:**
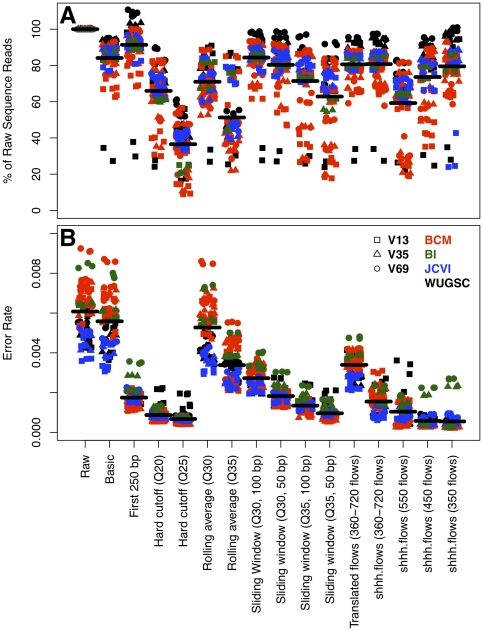
Effect of various sequence culling, trimming, and de-noising strategies on the number of non-chimeric reads that were longer than 200 bp and their error rates using sequence data generated by sequencing the 16S rRNA gene sequence of the same mock community. The horizontal lines represent the average across all samples within that treatment.

**Figure 2:**
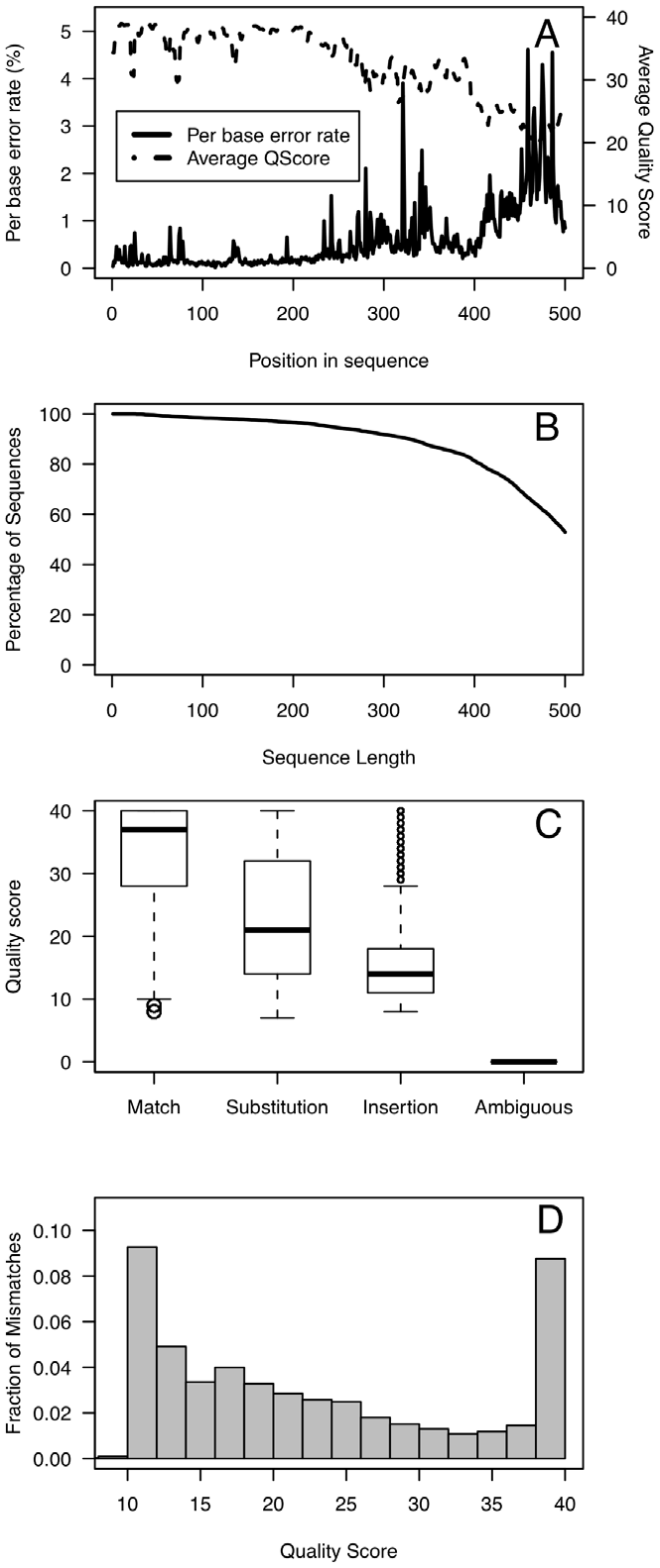
A typical error profile (accession SRX020131, V35, replicate 1) generated by sequencing the mock community without any sequence curation measures.

We first assessed the effects of PCR and sequencing-generated polymorphisms on the error rate of non-chimeric sequences. The average raw error rate across all regions, replicates, and runs was 0.0061 (standard deviation = 0.0013; Fig. 1B). We compared the intra-run error rate to the overall error rate and observed that 9 of the 10 sequencing runs had a significantly different mean from the overall error rate (all p<0.05). We also observed that the V69 region had a significantly higher error rate (0.0066; sd = 0.0015) compared to the V13 and V35 regions (0.0058; sd = 0.0012; p = 0.008). Sequencing errors accumulated toward the distal end of the sequence and the average Phred quality scores mirrored the observed errors along the length of the sequence (Fig. 2A). The insertion and deletion rates were 0.0024 (standard deviation = 0.0006) and 0.0019 (sd = 0.0008), respectively. Substitutions were less common and occurred at a rate of 0.0016 (sd = 0.0003). Finally, ambiguous base calls occurred at a rate of 0.0002 (sd = 0.0001). Ambiguous base calls, insertions, substitutions, and matches showed a clear association with the quality score for the base call (Fig. 2B). Interestingly, the distribution of quality scores for substitutions was bimodal suggesting that the lower quality substitutions were sequencing errors and the higher quality substitutions were due to PCR artifacts or chimeras whose parent sequences were from different operons in the same genome and were not detected by our approach because they were less than 3 bp different from each other (Fig. 2C).

### Identifying features of low quality sequences

Based on these general characteristics we sought to identify traits that were associated with low-quality sequences. First, we calculated the error rate of sequences that had between 0 and 2 mismatches to the barcode sequence and between 0 and 3 mismatches to the primer sequence. The error rate of sequences with 0 or 1 mismatches to the barcode had an error rate of 0.0056 (sd = 0.0013) and the sequences with 2 mismatches to the barcode had an error rate of 0.0101 (sd = 0.0118; p<0.001). Similarly, sequences that had two or fewer mismatches to the primer had an error rate of 0.0056 (sd = 0.0013), whereas sequences with three mismatches to the primer had an error rate of 0.0073 (sd = 0.0063; p = 0.004). Second, sequences that had one or more ambiguous base call had an error rate of 0.0111 (sd = 0.0014) and those without ambiguous base calls had an error rate of 0.0058 (sd = 0.0013; p<0.001). Third, sequences shorter than 200 bp had an error rate of 0.0163 (sd = 0.0053) and those longer than 200 bp had an error rate of 0.0059 (sd = 0.0014; p<0.001). Fourth, sequences that had homopolymers longer than 8 nucleotides (i.e. a string of consecutive and identical nucleotides) had an error rate of 0.0663 (sd = 0.0336) and those with homopolymers of 8 nucleotides or shorter had a significantly lower error rate of 0.0061 (sd = 0.0013). Finally, in each dataset there were sequences that did not align to the expected region of the 16S rRNA gene and were removed because they were clearly of dubious quality. Based on these results, we decided to cull sequences with more than 1 mismatch to the barcode, 2 mismatches to the primer, had an ambiguous base call, were shorter than 200 bp, had a homopolymer longer than 8 bp and that aligned to the incorrect region within the 16S rRNA gene. Based on these criteria the overall error rate decreased to 0.0056 and resulted in removing, on average, 15.9% of the sequences (sd = 10.9; Fig. 1, “Basic”).

### Trimming of sequences

**Figure 3:**
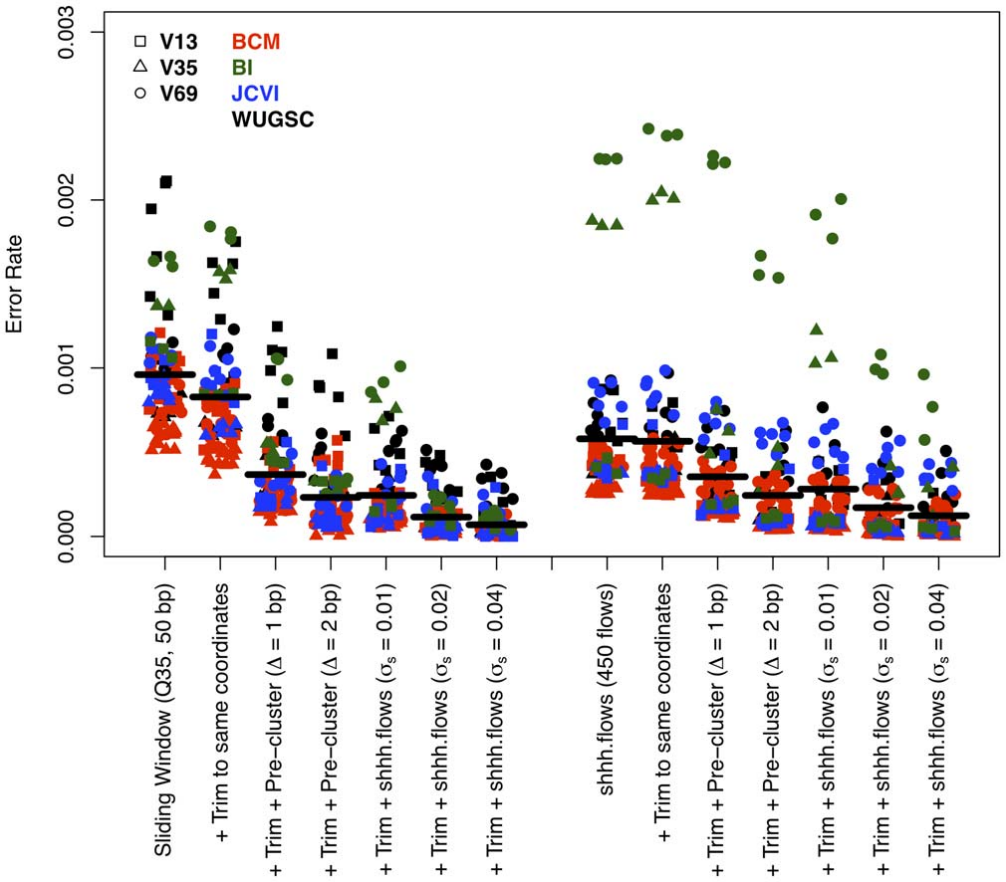
Effect of trimming sequences to the same alignment coordinates and using the pre-clustering or shhh.seqs algorithm on sequencing error rates when used within the sliding window and shhh.flows pipelines. The horizontal lines represent the average error rate across all samples within that treatment.

The association between error rates and quality scores shown in Fig. 2 suggested that if it were possible to identify break points where quality score criteria were no longer met, we could then trim sequences to those break points and reduce the overall error rates. First, we used a naïve approach based on the data presented in Fig. 2, which showed substantially lower error rates in the first 250 bp. Trimming sequences to 250 bp reduced the overall error rate to 0.0017 (Fig. 1B, “First 250 bp”). This approach was not ideal because it would not translate well to other platforms and might still allow base calls to pass that were of low quality. Instead, we sought an approach using the quality scores that could replicate this error rate. First, we implemented a hard cutoff by trimming each sequence at the first base that had a quality score below 20 or 25. This reduced the error rate by an average of 6.2 to 8.0-fold when compared to the Basic approach. Although use of a hard cutoff at a quality score below 25 reduced the error rate to 0.0007, it also reduced the number of non-chimeric sequence reads longer than 200 bp by 63.5% (Fig. 1, “Hard cutoff”). Second, we calculated the rolling average of the quality scores starting at the first base and trimmed the sequences when the average quality score dropped below 30 or 35 (Fig. 1, “Rolling average”). Neither threshold performed better than merely keeping the first 250 bp of each sequence. Third, we used a sliding window approach where the average quality score within a 50 or 100-bp window was calculated and when the average dropped below 30 or 35, the sequence was trimmed (Fig. 1, “Sliding window”). Requiring an average quality score of 35 over a 50 bp window resulted in error rates and average total number of sequences that were not meaningfully different from those observed using the sharp cutoff with a quality threshold of 20 (mean = 0.0010; sd = 0.0003). Based on our goal of retaining as many sequences as possible that were longer than 200 bp after removing the barcode and primer, while maintaining the lowest possible error rate, we settled upon using a sliding 50-bp window with an average quality score of 35 within the window. This was approach was selected because the resulting distribution of the number of sequences was broader than we observed using the sharp cutoff approach (Fig. 1B). Finally, because the 16S rRNA gene does not evolve uniformly over its length and the sequences resulting from this trimming algorithm generated sequences of varying length, it was necessary to further trim the sequences to a region where the average sequence was 200 bp long and all of the sequences started and ended in the same alignment positions [33], [34]. This trimming further reduced the error rate to 0.0008 (sd = 0.0004; Fig. 3) and insured that evolutionarily consistent regions were being compared.

### Denoising of flowgrams

We also explored the use of the PyroNoise algorithm, which reduces the sequencing error rate by correcting the original flowgram data using an expectation-maximization algorithm. We re-implemented this algorithm in the mothur software package as shhh.flows to take advantage of accelerated clustering algorithms and to make the algorithm more accessible to other researchers. For all of our analyses we removed any sequence with more than one mismatch to the barcode or more than two mismatches to the primer. Next, we used the Roche-454 determined quality cutoffs to identify those flowgrams that contained between 360 and 720 flows, as suggested in the PyroNoise documentation. Translation of these uncorrected flowgrams to DNA sequence resulted in an average error rate of 0.0034 (sd = 0.0006; Fig. 1, “Translated flows”). After processing these flowgrams through shhh.flows the error rate dropped to 0.0016 (sd = 0.0006; Fig. 1, “shhh.flows”). Based on our observation that sequence quality dropped after 250 bp, we trimmed all of the flowgrams to 350, 450, or 550 flows and processed the flowgrams through shhh.flows; after removing the barcode and primer, the resulting sequences were approximately 200, 250, or 300 bp long, respectively. Trimming of flowgrams to 350 or 450 flows resulted in an error rate of 0.0006 (sd = 0.0005); however, when we trimmed the flowgrams to 550 flows the average error rate increased to 0.0010 (sd = 0.0006). There were 7% fewer reads when the flowgrams were 450 flows long compared to trimming the flows to 350, but the sequences were approximately 30% longer. Compared to the output of the sliding window approach, the denoised flowgrams that were 450 flows long had a small improvement in average error rate and were approximately 50 bp longer. In addition, there were an average of 17.0% more sequences using shhh.flows than by using the sliding window. Although the shhh.flows approach is superior to the quality score-based trimming approach, we decided to pursue the sequences trimmed to 450 flows for our subsequent analysis in parallel to those obtained using the sliding window approach because of the high computational effort required for the shhh.flows approach.

### Denoising of sequences

We used two recently proposed approaches to remove lingering PCR amplification and sequencing errors. First, we modified the single-linkage pre-clustering algorithm proposed by Huse and colleagues [13], which joined sequence frequencies of sequences that were within a specified distance of each other assuming that the more abundant sequence was correct. Their original method used a distance matrix as the input; to save computational effort, we performed the clustering on the actual aligned sequences and counted the number of insertions, deletions, and substitutions between pairs of sequences. When we used the output of the quality trimming algorithm and the output of shhh.flows, the error rate for both approaches dropped to 0.0004 (sd = 0.0004) and 0.0002 (sd = 0.0002) when we allowed 1 or 2 mismatches to the more abundant sequence, respectively (Fig. 3). One limitation of this method is the risk of allowing too many mismatches, which would then limit the ability to cluster sequences into OTUs at fine-scale distance levels. For example, 2 mismatches between a rare and abundant sequence over 200 bp represents a 1% difference; however, if two rare sequences are each 2 bp different from the abundant sequence, they can be up to 4 bp or 2% different from each other. Allowing up to 3 mismatches over 200 bp would allow sequences that are as much as 3% different from each other to be pre-clustered making it difficult to resolve OTUs at the common 3% cutoff. Based on these observations, we recommend allowing 1 mismatch for every 100 bp of sequence data. Second, we implemented the SeqNoise algorithm in mothur as shhh.seqs. This algorithm is similar to shhh.flows except that the inputs are sequences and a model that describes rates of substitutions and homopolymeric insertions and deletions [24]. This approach uses the parameter σ_S_ to modulate the variation differences between the corrected sequence and the observed sequences. When we applied this algorithm to the data processed by the quality trimming approach using σ_S_ values of 0.01, 0.02, and 0.04 we observed error rates of 0.0002 (sd = 0.0002), 0.0001 (sd = 0.0001), and 0.0001 (sd = 0.0001), respectively; when applied to the output of shhh.flows we observed error rates of 0.0003 (sd = 0.0004), 0.0002 (sd = 0.0002), and 0.0001 (sd = 0.0002), respectively (Fig. 3). The reduction in error rates is offset by the challenge that as σ_S_ increased, the median maximum difference between the idealized sequence and the corrected sequences was 4, 6, and 11 bp, for the three σ_S_ values, respectively. Considering the possible loss of resolution for OTUs defined by small distances when using larger σ_S_ values, we favor the use of an σ_S_ value of 0.01. Since the pre-clustering approach gives the investigator the ability to directly set the maximum difference between sequences and the error rate was comparable to those generated by shhh.seqs, the remainder of our study uses data processed using the pre-clustering approach with a maximum difference of 2 bp between the abundant and more rare sequence.

### Contribution of chimeras

To this point, our results have measured the sequencing error rate independent of the chimeras known to populate sequence collections. Because we knew the actual sequences and the potential chimeras they could form, we were able to identify those sequences that were chimeric. Using the sequencing runs originating from BCM, BI, and JCVI, we found that 5.5% (sd = 1.4) of the raw sequence reads were chimeric. In contrast, on average, 15.9% (sd = 5.5) of the raw sequence reads from runs originating at the WUGSC were chimeric (p<2×10^−16^); there was no significant difference in rate of chimerism between regions (p = 0.84). Using the sliding window approach and pre-clustering, we found that the overall chimera rate dropped to an average of 2.3% (sd = 1.6%) with the BCM, BI, and JCVI sequencing centers having an average chimera rate of 1.9% (sd = 0.4%) and WUGSC having an average chimera rate of 4.2% (sd = 2.4%).

### Chimera removal

We explored several chimera removal strategies using chimera.slayer, Perseus, and Uchime. To measure the specificity and sensitivity of these three approaches, we only used a single representative of each sequence that emerged from the sequence trimming and denoising approaches. We decided to place chimera removal at the end of the overall sequence curation pipeline since at that point in the pipeline we had the greatest confidence in the abundance of each sequence type. We first tested the ability of chimera.slayer and Uchime to identify chimeras using a chimera-free reference database (i.e. the Gold reference set [21]; Table 1); Perseus cannot be used with a reference database. For both sequence curation pipelines, Uchime provided approximately a 10-percentage point greater sensitivity over chimera.slayer and little difference in specificity. We next tested the ability of chimera.slayer, Uchime, and Perseus to identify chimeras without a reference database. This approach assumes that more abundant sequences are less likely to be chimeric sequences and can thus be used as the reference dataset [23]. With this database-independent approach, Uchime and Perseus outperformed chimera.slayer and Uchime and Perseus had comparable sensitivity and specificity (Table 1). When we compared the database-dependent and –independent chimera checking methods, using Uchime in a database-dependent manner performed slightly better than the database-independent approaches (Table 1). Regardless, we decided to pursue the database-independent approach. This choice was made because the Gold reference collection only contains 16S rRNA gene sequences from cultured bacteria and was not expected to perform as well on real samples that contained as yet uncultured bacteria and archaea. Although the differences between Uchime and chimera.perseus were minimal, we decided to use Uchime for the remainder of this study because it had a lower false discovery rate and because of the previously reported faster execution times [32]. After using the database-independent implementation of Uchime, 34.6 (sd = 8.5) and 24.5% (sd = 10.8) of the remaining unique sequences were chimeric (i.e. false negative rate) and when the frequency of redundant sequences were included we found that 0.9% and 1.2% of the sequences were chimeric by the sliding window and shhh.flows pipelines, respectively.

**Table 1:** Ability of chimera.slayer, Uchime, and chimera.perseus to detect reliably chimeras within the sliding window and shhh.flows pipelines when using the Gold sequence collection or the dataset itself as the reference.

Sequence curation pipeline	Chimera detection algorithm	Database	Sensitivity^a^^,^ b	Specificity	False discovery rate	Initial % chimeras	Final % chimeras
Sliding window	chimera.slayer	Gold	68.7 (11.8)	95.0 (3.4)	3.7 (3.4)	67.8 (11.9)	39.6 (8.4)
		Self	66.5 (11.8)	94.2 (3.7)	4.4 (4.1)	67.8 (11.9)	41.4 (9.3)
	Uchime	Gold	79.2 (8.0)	93.7 (3.7)	4.1 (3.3)	67.8 (11.9)	30.6 (8.7)
		Self	74.5 (10.5)	94.4 (3.7)	4.0 (3.7)	67.8 (11.9)	34.6 (8.5)
	chimera.perseus	Self	76.0 (5.9)	91.6 (5.0)	5.6 (4.9)	67.8 (11.9)	34.8 (10.6)
shhh.flows	chimera.slayer	Gold	79.7 (7.7)	95.9 (2.6)	1.9 (1.2)	71.8 (7.3)	33.8 (13.2)
		Self	79.5 (7.1)	94.4 (3.6)	2.5 (1.4)	71.8 (7.3)	34.2 (12.3)
	Uchime	Gold	89.6 (6.3)	93.7 (3.9)	2.6 (1.7)	71.8 (7.3)	20.8 (11.3)
		Self	87.3 (5.8)	93.8 (4.0)	2.6 (1.6)	71.8 (7.3)	24.5 (10.8)
	chimera.perseus	Self	87.4 (6.8)	92.7 (4.3)	2.9 (1.5)	71.8 (7.3)	24.4 (12.4)

a All values in this table were calculated using unique sequences.

b The standard deviation is given in parentheses.

### Overall patterns in error rates and effects on clustering into OTUs and phylotypes

Next, we were interested in the number of OTUs and phylotypes that were observed using both pipelines after optimizing the pipelines based on error rates. First, we used the average neighbor clustering algorithm to assign sequences to OTUs at a distance threshold of 0.03 [34]. Using reference sequences of the same length and the same region, we expected to observe between 17 and 19 OTUs. Among the unprocessed sequence collections, the median number of spurious OTUs was 176. Using the shhh.flows pipeline we observed fewer spurious OTUs for the V13 and V69 sequence collections than we did using the sliding window pipeline and the number of spurious OTUs for the V35 sequences were comparable by both approaches (Table 2). Because we were not able to identify all of the chimeras in the sequence collections using Uchime, we hypothesized that many of the spurious OTUs were chimeric. Indeed, removing the true chimeras that were not detected by Uchime (see methods for description of detecting true chimeras) reduced the number of spurious OTUs considerably (Table 2). Next, we assigned sequences to genus-level phylotypes using the naïve Bayesian classifier trained on the RDP's taxonomic outline and implemented in mothur [34], [35]. Again, using the reference sequences that corresponded to each region and sequence collection we expected to observe either 17 or 18 genera per sample. Among the unprocessed sequence collections, the median number of spurious genera was 37. Once sequences were processed by either pipeline, the median number of spurious genera per sample varied between 1 and 9 (Table 2). When the non-detected chimeras were removed, the median number of spurious genera varied between 1 and 5. The number of spurious genera was comparable between the two pipelines (Table 2). Even though the sequencing error rate was reduced by 30-fold it still was not possible to obtain the expected number of OTUs and genera.

**Table 2:** Median number of spurious OTUs and genera identified in mock community sequence collections after removing chimeras identified using Uchime and removing all true chimeras within the two pipelines.

Region	Pipeline (Average error rate)	Chimera Removal	Median difference from number of expected OTUs	Median difference from number of expected genera
V13	Basic	Complete	168	11
	Sliding window (0.0004)	Partial	40	1
		Complete	23	1
	shhh.flows (0.0001)	Partial	10	2
		Complete	7	2
V35	Basic	Complete	156–157	36–37
	Sliding window (0.0001)	Partial	10	4–5
		Complete	5	1
	shhh.flows (0.0001)	Partial	11–12	5–6
		Complete	4	2
V69	Basic	Complete	220–221	51–52
	Sliding window (0.0002)	Partial	21	8–9
		Complete	9–10	5
	shhh.flows (0.0001)	Partial	12	4
		Complete	12	4

### Controlling for uneven sampling

We observed that the number of sequences obtained for a sample had a strong correlation to the number of observed OTUs (e.g. R = 0.50 by shhh.flows) and a weak correlation to the number of observed genera (e.g. R = 0.30 for shhh.flows); a similar result has been previously observed [14]. By randomly selecting sequences from each sample processed by shhh.flows so that each sample had the same number of sequences (n = 6,659 for V13, n = 4,615 for V35, and n = 3,129 for V69) we observed a median of 5, 4, and 3 spurious OTUs and 1, 2, and 1 spurious genera for the V13, V35, and V69 regions. Standardizing sample sizes is occasionally used because numerous alpha and beta diversity metrics are sensitive to sampling effort; our analysis underscores the need to standardize the number of sequences per sample because of the sensitivity of the number of spurious OTUs and genera to sampling effort.

### Biases related to PCR amplification

**Figure 4:**
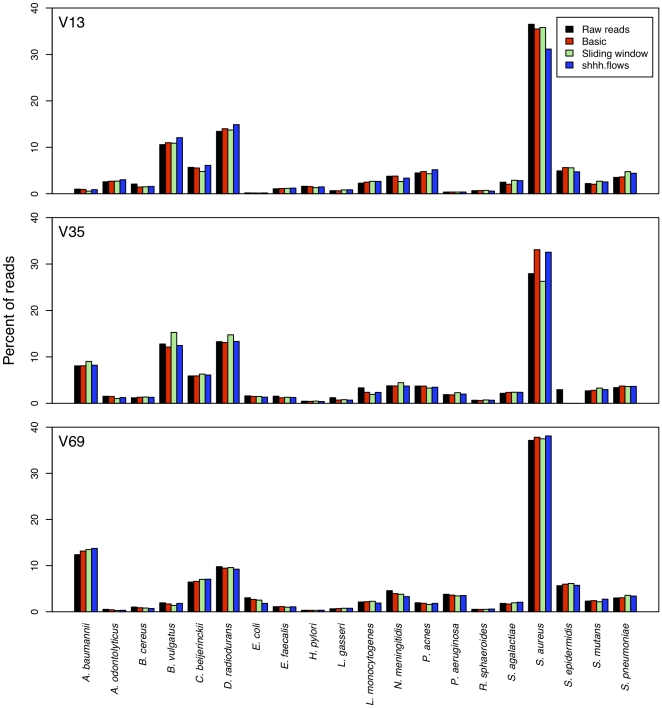
Differences in relative abundance of each sequence type in the raw unprocessed reads, following the Basic sequence curation steps, and at the end of the two pipelines. Each bar represents the average relative abundance for 30 sequencing collections.

Although there are numerous possible sources of bias in generating sequence data, in this study we were able to address those resulting from PCR amplification. Fig. 4 clearly shows that the distribution of relative abundances among the raw reads was skewed. Since replicate copies of the mock community were not independently generated, it was not possible to ascertain what fraction of the skew is due to error in assembling the DNAs or PCR bias. Table 3 lists the primer sequences that were used to PCR amplify the three regions and their homology to members of the mock community. Although each of these primers would be able to amplify DNA from all of the bacterial genomes, those sequences that did not have perfect homology to the primer amplified at a lower efficiency than those with perfect homology. The primers used to amplify the V13 region had the most dramatic effects on the relative abundance compared to the other regions. For instance, *A. baumannii*, *E. coli*, and *P. aeruginosa* V13 sequences were nearly undetectable in the raw sequence pool. This result can be explained by a one base mismatch in the middle of the forward primer sequence, which is a common variant among many bacteria and can be recovered using a degenerate form of the primer [36]. Interestingly, most of the differences observed in the relative abundances of each sequence type in the raw reads could be explained by mismatches to primers suggesting that amplification bias was not significant.

**Table 3:** The primer sequences used to amplify the three regions of the 16S rRNA gene and their homology to the 16S rRNA gene sequences represented in the mock community.

Primer^a^	Organism (fraction of operons^b^)	Sequence^c^
V13F	Consensus (94/113)	AGAGTTTGATCCTGGCTCAG
	*A. baumannii* (5/5)	...........A........
	*B. vulgatus* (1/7)	.............A......
	*E. coli* (7/7)	...........A........
	*P. aeruginosa* (4/4)	...........A........
V13R	Consensus (108/113)	ATTACCGCGGCTGCTGG
	*P. acnes* (3/3)	..C..............
V35F	Consensus (111/113)	CCTACGGGAGGCAGCAG
V35R	Consensus (109/113)	CCGTCAATTCMTTTRAGT
	*H. pylori* (2/2)	.....T............
V69F	Consensus (93/113)	ACGCGAAGAACCTTAC
	*B. vulgatus* (7/7)	......G.........
	*H. pylori* (2/2)	..A.............
	*P. acnes* (3/3)	.....T..........
	*R. sphaeroides* (6/6)	.....C..........
V69R	Consensus (107/113)	TACGGYTACCTTGTTAYGACTT
	*B. cereus* (1/12)	....A.................
	*D. radiodurans* (3/3)	....A.................

a “F” corresponds to the forward PCR primer and “R” corresponds to the reverse PCR primer.

b *M. smithii* (2 operons) had very low homology to all three primer pairs and is not depicted here.

c All primers are written in the 5′ to 3′ orientation. The complete primers were synthesized to have a barcode and adapter primer at the 5′ end of the reverse primer and an adapter primer at the 5′ end of the forward primer. Sequencing was performed using the adapter downstream of the reverse primer.

### Biases related to analysis pipeline

There were few obvious biases observed in the relative abundance of each sequence type as processed by the sliding window and shhh.flows pipelines. Comparing the relative abundances in the raw reads to those that were obtained after the “Basic” sequence curation revealed a large difference in the V35 data for *S. aureus* and *S. epidermidis* by both pipelines. This is explained by the fact that the first 260 bp that were sequenced in this region were identical for these two organisms. Unique to the sliding window pipeline, we observed a large decrease in the relative abundance of *S. aureus* in the V35 data. Unique to the shhh.flows pipeline, we observed a large decrease in the relative abundance of *S. aureus* in the V13 data. There were no differences in rates of chimerism for this population or remarkable features of the pipeline that explained this bias, suggesting that the bias may be attributable to biases inherent in the ability of the sequencing platform to generate similar quality data across sequence types.

### Biases between sequencing centers

**Figure 5:**
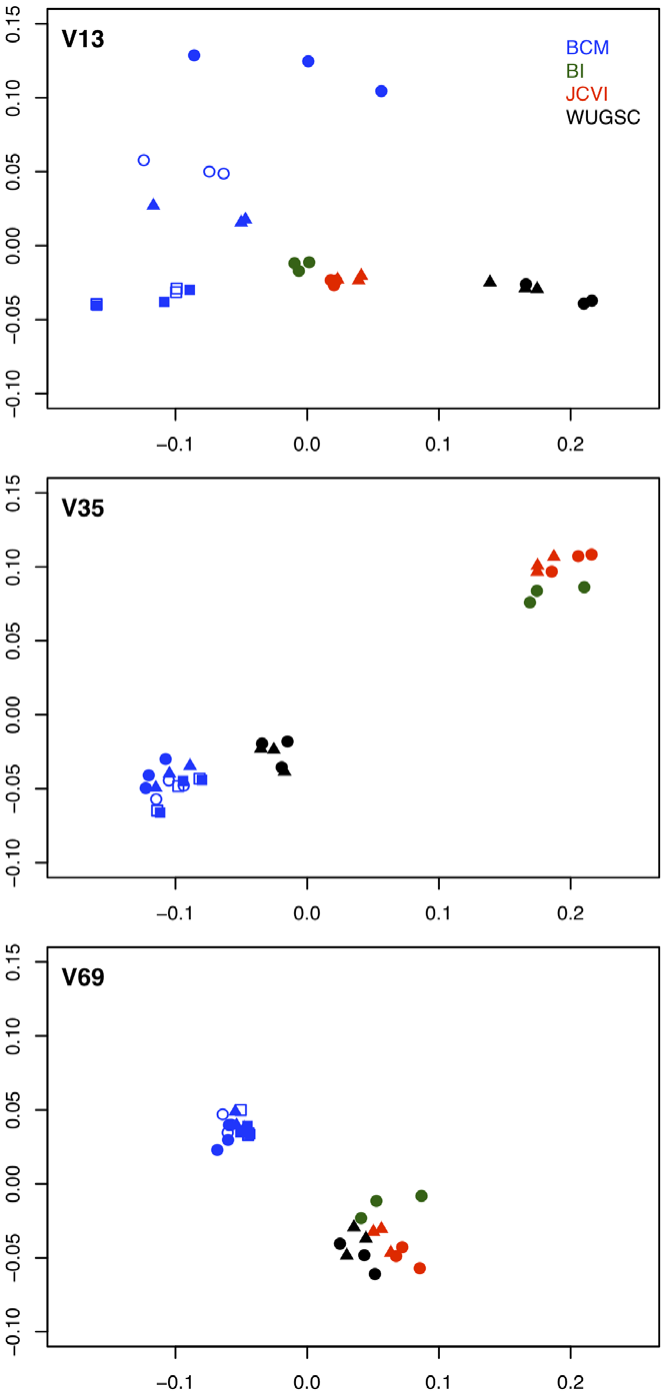
Non-metric multidimensional scaling (NMDS) plot generated using ThetaYC distances between mock community sequencing data after standardizing the number of sequences per sample. Points with the same color and shape originated from the same sequencing run.

**Figure 6:**
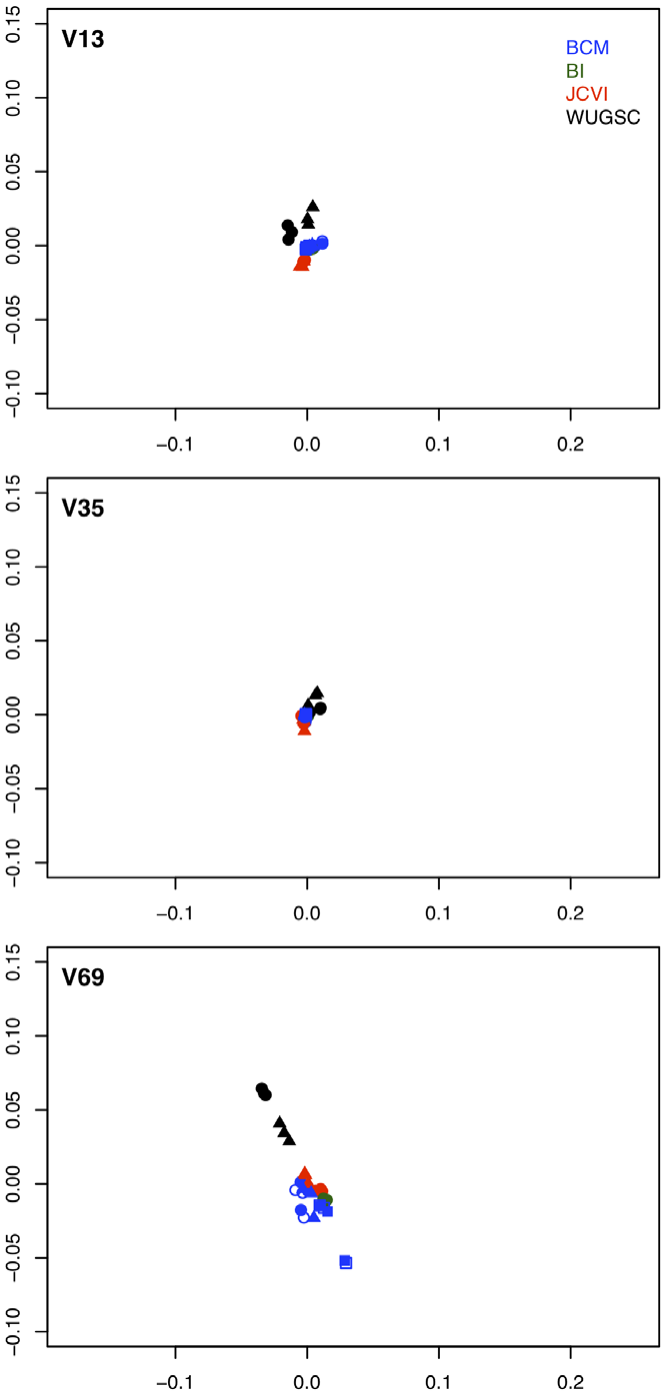
NMDS plot generated using ThetaYC distances between sequencing replicates of the same stool sample after standardizing the number of sequences per sample. Points with the same color and shape originated from the same sequencing run.

We generated a non-metric multidimensional scaling (NMDS) plot of distances calculated using the ThetaYC measure of community dissimilarity for each region after randomly sub-sampling the samples so that they would each have the same number of sequences (Fig. 5). Interestingly, the mock community samples clustered by sequencing center and then by sequencing run. To explore this effect further, we analyzed 16S rRNA gene sequences from a control DNA preparation from a single human stool sample that were generated in parallel to the 90 mock community sequence sets. We processed the 90 sequence collections generated from the same stool sample using the shhh.flows pipeline, removed any OTUs that were classified as being derived from a chloroplast or mitochondria, and standardized the number of sequences per sample to the smallest sequence collection for that region. We measured the alpha diversity of the stool sample using the observed richness and the inverse Simpson diversity index based on the frequency of OTUs and genera in the sequence collections. Within a region, the richness and diversity of OTUs and genera were comparable among the BCM, BI, and WUGSC samples and the richness and diversity were lower in the JCVI samples (Table 4). Variation between regions was expected because the number of sequences varied and the 16S rRNA gene does not evolve uniformly along its length [33]. Finally, we generated an NMDS plot to compare the samples using ThetaYC distances (Fig. 6). There was considerably less variation between samples using the same stool sample than there was with the mock community sample; however the samples still clustered by sequencing center and run. Although the difference in results observed between the stool sample and the mock community samples deserves further attention, we suspect this occurred because the stool sample was dominated by fewer taxa than the mock community and was thus not as affected by subtle differences in the experimental setup (e.g. thermalcycler and pipette calibration, pipettor error, reagent lot, primer concentration, etc.).

**Table 4:** The richness and diversity of OTUs and genera identified in the control stool sample sequence collections for each region and sequencing center.

Region	Number of sequences per sample	Center	Observed Richness (OTU; sd)	Inverse Simpson (OTU; sd)	Observed Richness (Genera; sd)	Inverse Simpson (Genera; sd)
V13	9,577	BCM	33.1 (4.1)	2.13 (0.08)	19.3 (2.6)	1.22 (0.01)
		BI	30.7 (1.5)	2.09 (0.02)	18.3 (0.6)	1.20 (0.01)
		JCVI	25.0 (2.8)	1.82 (0.04)	14.0 (2.5)	1.16 (0.01)
		WUGSC	37.2 (8.3)	2.30 (0.21)	19.5 (2.7)	1.48 (0.04)
V35	2,741	BCM	21.3 (2.7)	2.08 (0.04)	16.3 (2.3)	1.21 (0.02)
		BI	20.7 (1.5)	2.13 (0.02)	19.7 (1.5)	1.22 (0.01)
		JCVI	18.3 (2.3)	1.94 (0.04)	13.2 (1.7)	1.13 (0.03)
		WUGSC	25.2 (4.1)	2.35 (0.15)	16.8 (3.4)	1.39 (0.07)
V69	4,294	BCM	43.9 (4.4)	3.88 (0.43)	23.7 (3.3)	1.94 (0.24)
		BI	39.0 (1.0)	4.14 (0.50)	24.0 (2.6)	2.08 (0.07)
		JCVI	40.0 (2.3)	3.64 (0.25)	19.2 (3.1)	1.88 (0.19)
		WUGSC	37.3 (5.2)	2.82 (0.25)	18.8 (2.8)	1.59 (0.11)

### Analysis of human microbiome data

**Figure 7:**
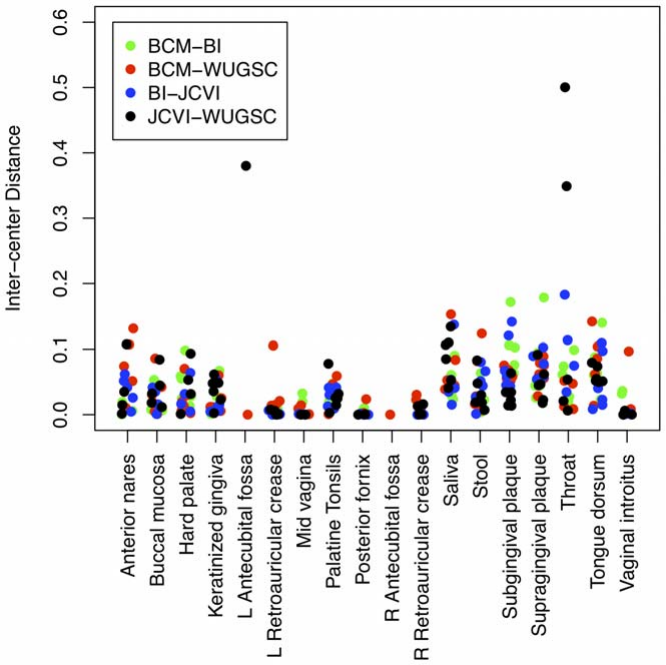
Pairwise ThetaYC distances between different DNA samples that were PCR amplified and sequenced at two different sequencing centers using the V13 region. The uneven distribution of points across body sites is due to the inability of the sequencing centers to generate more than 2,000 sequence reads for that sample.

To understand how the magnitude of the inter-center variation we observed with the control stool sample translated to other body sites, we re-analyzed a dataset that characterized 15 or 18 body sites from 12 men and 12 women, respectively. The study was structured such that the DNAs were all extracted at BCM and the DNAs were then sent to two of the four sequencing centers where PCR and sequencing were performed. We re-analyzed the sequence data using the shhh.flows-based pipeline and sub-sampled sequences within each body site so that the same number of sequences were used for each sample. We observed broad inter-center variation at most body sites with most differences being less than 0.20 (Fig. 7). For the stool samples, the interquartile range of pairwise distances varied between 0.0143 and 0.0607 and between 0.0092 and 0.0394 for the V13 and V35 regions, respectively. In comparison, the interquartile range of distances between the 30 technical replicate sequencings of the control stool sample varied between 0.0023 and 0.0174 and 0.0013 and 0.0098 for the V13 and V35 regions, respectively. In general, samples with lower diversity also had lower inter-sample variation (e.g. Mid vagina vs. Stool). Because of the small number of samples that were analyzed by each pair of centers, it was not possible to assess whether there were systematic center-based biases. Regardless, it is clear that intra- and inter-center variation can have a significant impact on the ability to detect subtle differences between treatments.

## Discussion

A fundamental problem with the use of next generation sequencing is that the technologies have been developed primarily for resequencing, mapping, and genome assembly, which require relatively low data quality compared to the single read analysis required of 16S rRNA gene sequencing. In addition, further sequencing of genomes will get an investigator closer to the truth, whereas we have shown that additional sequencing for single read analysis increases the number of artifacts and may exacerbate biases via steps in the data generation protocol. Here we showed that the presence of chimeric 16S rRNA gene sequences and sequencing errors have a significant impact on the biodiversity reflected in microbial ecology studies. To correct for these sources of error we have proposed a number of solutions that can be tailored to a particular analysis. We have successfully reduced the sequencing error rate by 30-fold and the number of chimeric sequences by 10-fold. The reduction in sequencing error rate is at least an order of magnitude lower than those reported in previous studies.

Although the number of replicate mock communities and control samples analyzed in this study is beyond the capacity of most laboratories, this analysis has demonstrated the value of internal controls in microbial ecology studies. We have shown that even when sequencing centers follow the same procedures there is variation between and more importantly, within centers. The inclusion of a mock community sample on each sequencing run can be used to calculate the rate of chimerism, sequencing error rate, and drift in the representation of a community structure. Tailoring the richness and genetic diversity of a mock community to the samples of interest (e.g. stool, skin, soil, water, etc.) would be a useful resource for any laboratory performing microbial ecology research. Similarly, re-sequencing a control sample on every sequencing run is a useful datum to include if the control sample is representative of the biodiversity and complexity of the other samples being analyzed. We have shown that even when following a proscribed and detailed protocol, intra- and inter-sequencing center variation can be significant. It is likely that insidious variation in thermalcycler calibration, reagent concentrations, and other factors are the cause of this technical variation. That this variation was observed using DNAs that were processed by one individual suggests that dividing the labor among many individuals could be a source of additional technical variation. Technical variation could hinder the ability to detect real differences and perhaps more worrisome is the potential for batch effects that have confounded genome-wide association studies (GWAS) [37]. In fact, the full HMP cohort of 300 healthy individuals must contend with many issues similar to those encountered in GWAS. Population structure in the broader HMP study, including race and ethnicity, was confounded with geographical location. Geographical location, likewise, was confounded with clinical sampling, as individuals were recruited uniformly in Houston at BCM or in St. Louis at WUGSC. Finally, clinical sampling was in turn confounded with sequencing center, as all of the St. Louis samples were sequenced at WUGSC while the Houston samples were divided among BCM, JCVI, and BI. These factors together combine to produce at least five-fold more OTUs that exhibited significant associations with the subject's city of origin and the sequencing center than with any other clinical variable (Personal communication: HMP consortium). Incorporation of improved experimental design and development of correction schemes will be helpful as 16S rRNA gene sequencing surveys grow to encompass large, structured populations. Furthermore, it is critical that meta-analyses that hope to use this dataset as a reference take into account these non-biological sources of variation.

Our study has primarily addressed the effects of artifacts generated by PCR and sequencing; however, our analysis and that of numerous others suggests that biases in the representation of the 16S rRNA gene pool may confound interpretation of these microbial communities. Because a single aliquot of the mock community was re-sequenced instead of multiple aliquots of the same mock community it was not possible to directly measure the magnitude of PCR-related biases relative to the ratios of the different organisms in the mock community. Regardless, interpretation of microbiome data in light of relative differences in abundance and the use of complementary methods should reduce the effects of such biases and lingering PCR and sequencing artifacts. Although it may not be possible to ascertain specific values for alpha and beta diversity measures, changes in the observed parameter (e.g. richness or relative abundance) can be used to indicate general changes in the community.

Any microbial community analysis is only as good as the underlying biological question, study design, DNA extraction method, PCR conditions, sequencing, and bioinformatic analysis. Much of the recent debate over the scope of the “rare biosphere” has focused on the sequencing aspect of this pipeline without considering the biases and artifacts introduced at the other steps. In fact, all methods have their own strengths and limitations and introduce unique biases. Next generation sequencing is but one of many powerful tools at our disposal for relating changes in microbial community structure with changes in health; however, it is critical that we consider each step in the analysis and use multiple methods to triangulate on our biological questions.

## Materials and Methods

### mother

All analyses described in the current study were performed within version 1.22 of the mothur software package [38]. We implemented the PyroNoise algorithm in mothur as the shhh.flows command [24]. shhh.flows makes use of mothur's accelerated clustering algorithms, has a significantly improved user interface, and because mothur runs on multiple platforms, it is no longer constrained to Unix-based operating systems. Our implementation of ChimeraSlayer within mothur as chimera.slayer provides for faster execution, parallelization, the ability to use any reference alignment, and an improved interface [21]. We also modified chimera.slayer to identify chimeras without the use of a stand-alone reference database by treating the more abundant sequences in a dataset as the reference. Within mothur, we created a wrapper for the original Uchime source code, which was implemented as chimera.uchime. Finally, we re-implemented the PerseusD code in mothur as chimera.perseus [32]. The only difference between our implementation and the original was to use a star alignment instead of MAFFT to align the query sequence to its two putative parents. The concordance between what the two implementations identified as chimeric was generally above 99%. shhh.flows, shhh.seqs, the Bayesian classifier, and chimera.slayer were C++ translations of the original C, Java, and Perl programming code and generated identical output of the original software when benchmarked using test datasets provided by the original developers. Those interested in following the pipelines described in this study can follow the tutorial on the mothur website (http://www.mothur.org/wiki/Schloss_SOP).

### Identification of chimeras and sequencing errors

All reference and *de novo* sequences were aligned to a 50,000-column wide SILVA-based reference alignment using mothur's NAST-based aligner [39], [40]. We identified chimeras in the mock community data by calculating the number of mismatches between each sequence and all possible two-parent chimeras that could be generated by the reference sequences. If a sequence was at least three bases more similar to a chimera of reference sequences than to a single reference sequence, then it was considered chimeric [24]. Sequences that met this criterion were excluded from the calculation of error rates. The error rate of non-chimeric sequences was calculated by calculating the distance between the query sequence and each reference sequence. The reference sequence with the shortest distance to the query was considered the true reference sequence.

### Mock community

A single aliquot of the mock community was used throughout the sequencing effort analyzed in this study. This mock community represented 21 strains distributed among members of the Bacteria (n = 20) and Archaea (n = 1). Among the 20 bacterial sequences, there were 6 phyla, 10 classes, 12 orders, and 18 families and genera. The aliquot of mock community DNA was prepared by mixing genomic DNA from *Acinetobacter baumanii* (NC_009085), *Actinomyces odontolyticus* (DS264586), *Bacillus cereus* (AE017194), *Bacteroides vulgatus* (NC_009614), *Clostridium beijerinckii* (NC_009617), *Deinococcus radiodurans* (NC_001263), *Enterococcus faecalis* (NC_004668), *Escherichia coli* (NC_000913), *Helicobacter pylori* (NC_000915), *Lactobacillus gasseri* (NC_008530), *Listeria monocytogenes* (NC_003210), *Neisseria meningitidis* (NC_003112), *Propionibacterium acnes* (NC_006085), *Pseudomonas aeruginosa* (NC_002516), *Rhodobacter sphaeroides* (NC_007493, NC_007494), *Staphylococcus aureus* (NC_007793), *Staphylococcus epidermidis* (NC_004461), *Streptococcus agalactiae* (NC_004116), *Streptococcus mutans* (NC_004350), *Streptococcus pneumoniae* (NC_003028), and *Methanobrevibacter smithii* (NC_009515). Given the low homology between the three PCR primer pairs and the *M. smithii* 16S rRNA gene sequence, these sequences were rarely observed and have been omitted from the analysis of this study. The proportions of genomic DNAs added were calculated to have an equal number of 16S rRNA genes represented for each species; however, the original investigators did not verify the final relative abundances.

### DNA sequence data

All of the DNA sequences that were included in this study were obtained by following a common SOP (http://www.hmpdacc.org/doc/HMP_MDG_454_16S_Protocol_V4_2_102109.pdf). Of particular note is that the sequencing was performed by sequencing from the reverse PCR primer towards the forward primer. All sequence data is available through the HMP Data Analysis and Coordination Center (DACC; http://www.hmpdacc.org/HMMC) and the NCBI Short Read Archive (http://www.ncbi.nlm.nih.gov/sra).

### α and β Diversity measurements

All calculations were performed using mothur. α diversity was measured by counting the number of observed OTUs (i.e. observed richness) and using the reciprocal of the Simpson Index as described by Magurran [41]:
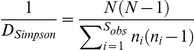
Where N is the total number of sequences sampled from the community, n_i_ is the number of sequences in the i^th^ OTU, and S_obs_ is the total number of OTUs. The reciprocal Simpson Index was selected because it represents the number of uniformly distributed OTUs that were required to have the same diversity as the actual community; thus giving it an easier biological interpretation compared to other indices (e.g. Shannon Index). β diversity was measured by using the θ_YC_ distance of Yue and Clayton [42]:
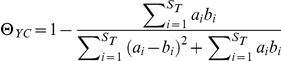
Where a_i_ and b_i_ are the relative abundances of the i^th^ OTU in communities A and B and S_T_ is the total number of OTUs observed in both communities. θ_YC_ measures differences in community structure and was selected because it weighs rare and abundant OTUs more evenly than other metrics such as Bray-Curtis or Morisita-Horn.
